# TRAIL-R1-Targeted CAR-T Cells Exhibit Dual Antitumor Efficacy

**DOI:** 10.3389/fmolb.2021.756599

**Published:** 2021-12-20

**Authors:** Yaru Nai, Li Du, Meiying Shen, Tingting Li, Jingjing Huang, Xiaojian Han, Feiyang Luo, Wang Wang, Da Pang, Aishun Jin

**Affiliations:** ^1^ Chongqing Key Laboratory of Basic and Translational Research of Tumor Immunology, Chongqing Medical University, Chongqing, China; ^2^ Department of Immunology, College of Basic Medicine, Chongqing Medical University, Chongqing, China; ^3^ Department of Breast Surgery, Harbin Medical University Cancer Hospital, Harbin, China; ^4^ Department of Endocrine Breast Surgery, The First Affiliated Hospital of Chongqing Medical University, Chongqing, China

**Keywords:** TRAIL-R1, CAR-T, apoptosis, cytotoxicity, third generation

## Abstract

Tumor necrosis factor–related apoptosis-inducing ligand receptor 1 (TRAIL-R1) has limited expression in normal tissues but was highly expressed in various types of tumors, making it an attractive target for cancer immunotherapy. Here, we utilized the single-chain variable fragment (scFv) from our previously identified TRAIL-R1–targeting monoclonal antibody (TR1^419^) with antitumor efficacy and produced the TR1^419^ chimeric antigen receptor (CAR) T cells. We characterized the phenotypes and functions of these CAR-T cells and found that the third-generation TR1^419^-28BBζ CAR-T cells exhibited greater target sensitivity and proliferative capability, with slightly higher PD-1 expression after antigen stimulation. Importantly, we found that the TR1^419^ CAR-T cells could induce TRAIL-R1–positive tumor cell death *via* a dual mechanism of the death receptor–dependent apoptosis as well as the T-cell–mediated cytotoxicity. Altogether, the TR1^419^ CAR-T cells could serve as a promising strategy for targeting the TRAIL-R1–positive tumors.

## Introduction

CAR-T cells have been proven to be promising modalities of adoptive cellular therapy (Hillerdal et al., 2014; June et al., 2018). Beneficial advances have been reported with the CD19-targeted CAR-T cells in the treatment of diffuse large B-cell lymphoma (DLBCL) (Kochenderfer et al., 2015; Lee et al., 2015; Turtle et al., 2016). However, there are still many encumbered challenges with the applications of CAR-T cells against solid tumors (Zhang, 2016; Guedan et al., 2018; Stoiber et al., 2019). CARs are composed of an antigen recognition domain of a single-chain variable fragment (scFv), hinge region, transmembrane domain, intracellular signaling region, co-stimulatory domain, and CD3ζ chain (Hillerdal and Essand, 2015; Du et al., 2019; Ramello et al., 2019). The scFv region is responsible for the binding of the antigenic target, and the co-stimulation domain plays an essential role in promoting the expansion and antitumor effects of CAR-T cells, while greatly related to the design of the scFv structure (Guedan et al., 2018). It has been reported that the phenotypic and functional optimizations of CAR-T cells largely rely on the design of the scFv and the corresponding second- or third-generation co-stimulatory domains (Savoldo et al., 2011; Golumba-Nagy et al., 2018; Sun et al., 2020).

One key to the clinical success of CAR-T therapy is the selection of an ideal target antigen, preferentially expressed on the surface of cancer cells at high levels (Tseng et al., 2020). TRAIL is a member of the tumor necrosis family (TNF) superfamily that interacts with its death receptors (TRAIL-R1/TR1/DR4) to induce apoptosis (Siegemund et al., 2016; Dufour et al., 2017). Importantly, the TRAIL receptor signaling–induced apoptotic cell death was found in a wide range of cancer cell types, but not in normal cells. So far, clinical trials with TRAIL-based therapy have been unsatisfactory due to the little antitumor efficacy as well as the high rate of resistance observed *in vivo* (Fu et al., 2012; Lv et al., 2020). In a previous report, we have presented a fully human monoclonal agonistic antibody targeting TRAIL-R1 (TR1^419^), which could strongly induce apoptosis in a wide range of TRAIL-R1**–**expressing cancer cell lines, in the presence of crosslinking antibodies (Jin et al., 2009; Jin et al., 2010). Considering the unique tumor specificity of TRAIL-R1 expression, we suspected that incorporating the TR1^419^ scFv region into a CAR may bring therapeutic efficacy for the CAR-T cells.

In the present study, we generated the second- and the third-generation TR1^419^ CAR-T cells. The results showed that the third-generation TR1^419^-28BBζ CAR-T cells had higher sensitivity to the target antigen and exhibited a better proliferative ability but had a higher level of PD-1 expression after antigenic stimulation. These CAR-T cells were shown to induce significant amount of target tumor cell death not only *via* TRAIL-R1–mediated apoptosis but also *via* CAR signal–induced cytolysis. This study suggests that TR1^419^ CAR-T cells could be a promising strategy targeting the TRAIL-R1–positive tumors, and the dual killing mechanism provides a novel optimizing strategy for the CAR design.

## Materials and Methods

### Cell Lines and Cell Culture Conditions

SW480, HCT116, K562, and Huh7 were obtained from ATCC. The human Jurkat cell line was maintained in our laboratory, which neither produced IFN-γ nor exerted effector killing functions. These cells were cultured in the RPMI 1640 medium (Gibco, Invitrogen, Carlsbad, CA) supplemented with 10% fetal bovine serum (FBS) (Biological Industries, ISR). Human breast cancer cell lines MDA-MB-231 and HS578T were obtained from ATCC and cultured in the Dulbecco’s modified Eagle medium (Gibco, Invitrogen, Carlsbad, CA) supplemented with 10% fetal bovine serum (FBS) (Biological Industries, ISR). The HEK-293T cell line was purchased from ATCC and maintained in a complete growth medium (Gibco, Invitrogen), containing with 10% fetal bovine serum (FBS) (Biological Industries, ISR), 2 mM L-glutamine (Gibco, Invitrogen), and 1 mM sodium pyruvate solution (Gibco, Invitrogen). All the aforementioned cells were cultured in a humidified atmosphere containing 5% CO_2_ at 37°C.

### Plasmid Construction and Lentivirus Preparation

The target TR1^419^ CAR consisted of TR1^419^ scFv, CD28, or 4-1BB co-stimulatory domains and a CD3ζ signaling domain (Gross et al., 1989; Irving and Weiss, 1991). They were obtained by overlap PCR amplification; PCR products were cloned into a lentivirus vector pWPXL and verified by enzymatic digestion and sequencing. In order to produce the lentivirus supernatant, 8 × 10^5^ HEK-293T cells were seeded in 6-well plates. After 24 h, 293T cells were transfected with pMD.2G encoding VSV-G envelope, pSPAX2 lentivirus plasmid, and the CAR-pWPXL plasmids by using the Xfect Transfection Reagent (Takara xfect™), according to the manufacturer’s instructions. The culture supernatants of 293T cells containing lentiviral particles were collected and filtered with a 0.45-μM filter at 48 h after transfection.

### Human CAR-T Cell Generation

Human peripheral blood mononuclear cell (PBMC) samples were obtained from healthy donors isolated by using MACSprep™ PBMC Isolation Kit (Miltenyi Biotec, Germany), according to the manufacturer’s instructions. Written informed consents were obtained from all volunteer. In brief, The PBMCs were activated for 48 h in 24-well tissue culture–treated plates (2 × 10^6^/well) with anti-CD3 mAb (Miltenyi, Biotec, 1 μg/ml) and anti-CD28 mAb (Miltenyi, Biotec, 1 μg/ml) in a complete medium containing 90% RPMI 1640 and supplemented with 10% FBS (Gibco), 200 U/ml IL-2, 25 mM HEPPES, 55 μM 2-M, 100 U/ml penicillin, and 100 μg/ml streptomycin. After 2 days, the 2 × 10^5^ activated T cells were infected with 250 μL of CAR-encoding lentiviral supernatant in a tissue culture–treated 24-well plate. After lentiviral infection for 24 h, the lentiviral supernatant was replaced with a fresh complete medium, and cell culture were maintained at 37°C with 5% CO_2_. On the 7th day after transfection, the T cells were collected for subsequent experiments.

### Flow Cytometry

For cell surface staining, the lentivirus-infected T cells were harvested and washed with phosphate-buffered saline (PBS) followed by incubation with 1ug/ml recombinant protein TRAIL-R1-Fc (R&D Systems, Minneapolis, MN) at room temperature for 20 min. After being washed, the cells were incubation with 1ug/ml APC-conjugated anti-human IgG secondary antibodies and other cocktail antibodies (Biolegend) at room temperature for 15 min in the dark. After being washed with PBS, the stained cells were analyzed by flow cytometry (BD Biosciences). The following monoclonal antibodies were used with the indicated subtypes: APC-labeled human IgG-Fc (clone HP6017), Percy5.5-labeled anti-CD4 (clone RPA-T4), Alex700-labeled anti-CD8 (clone HIT8a), BV421-labeled anti-CCR7 (clone G043H7), PE-labeled anti-CD45RO (clone UCHL1), AF488-labeled anti PD-1 (clone EH12.2H7), BV605-labeled anti-LAG-3 (clone 11C3C65), and BV785-labeled anti-TIM-3 (clone F38-2E2). All sample data analyzed was done on ≥10,000 events using the FlowJo V 10 data analysis software.

### Killing Activity Assay

The ability of CAR-T cells to kill tumor cells was determined *via* calcein AM (CAM, Dojindo) release–based cytotoxic cell assay (Jonsson et al., 1996). In brief, target cells were stained with 10 μM CAM for 30 min at 37°C, followed by terminating the reaction with addition of Dulbecco’s phosphate-buffered saline without calcium (DPBS) buffer. The target cells were washed five times with DPBS. Non-transduced T cells were used to normalize the percentage of CAR-positive cells. Then CAR-T cells and target cells were plated in a 96-well microplate at various effector/target (E:T) ratios at 37°C. After incubation for 6h, the 50 μl supernatant was transferred to 96-black plates to measure fluorescence intensity (FI) at 485 nm excitation and 520 nm emission wavelengths. Three parallel holes were set for each group. The percentage of cytotoxicity was calculated according to the following formula: lysis%=(test release−spontaneous release)/(maximal release−spontaneous release)×100%.

The 293T cell lysis by CAR-T cells was assessed by using real-time cell analysis (RTCA, ACEA)–based killing assay. One prior day to seed approximately 1 × 10^4^ 293T cells on the 16 E-plate, the cells were grown to mid-logarithmic growth phase. Non-transduced T cells were used to normalize the percentage of CAR-positive cells, and CAR-T cells were added to the cultures at indicated effector-to-target ratio. A RTCA DP analyzer was used to monitor real-time target cell growth, and the results were analyzed by RTCA software (Bilandzic et al., 2019). The percentage of cytotoxicity was calculated according to the following formula: lysis%=[1−(experiments/empty culture)]×100%.

### Enzyme-Linked Immunosorbent Assay

The CAR-T cells and target tumor cells were cocultured at s various E:T ratios in a 96-well plate for 24 h at 37°C. The culture supernatants were harvested and subjected using ELISA to detect IFN-γ and granzyme B, according to the manufacturer’s instructions. Every value represented the mean of triplicate wells.

### Tumor Cell Apoptosis Assay

CAR-T cells and target cells were incubated for 2 h at 37°C, and target cell apoptosis was assayed using an Annexin V/7-AAD Apoptosis Detection Kit (Biolegend), according to the manufacturer’s instructions. Target cell caspase-3 activity was detected used a caspase-3 activity detection assay kit (BD). In brief, the cells were washed twice with cold PBS, followed by fixing and permeabilizing in Cytofix/CytoPerm for 20 min on ice (BD Biosciences). Subsequent staining was performed with 1 × PermWash as the staining and wash buffer and incubated for 15 min at room temperature. Washed cells were analyzed with BD Celesta (BD Biosciences) software by flow cytometry.

### Statistical Analysis

Statistical analyses in this study were performed with GraphPad Prism software 8.0.2 version. Data were presented as mean ± SEM. When there were two experiment groups, Student’s t test was used or ANOVA analysis for multiple comparisons. *p* values < 0.05 were considered statistically significance.

## Results

### TR1^419^-28BBζ CAR-T Cells Exhibited Functional Advances Upon Antigen Stimulation

**FIGURE 1:**
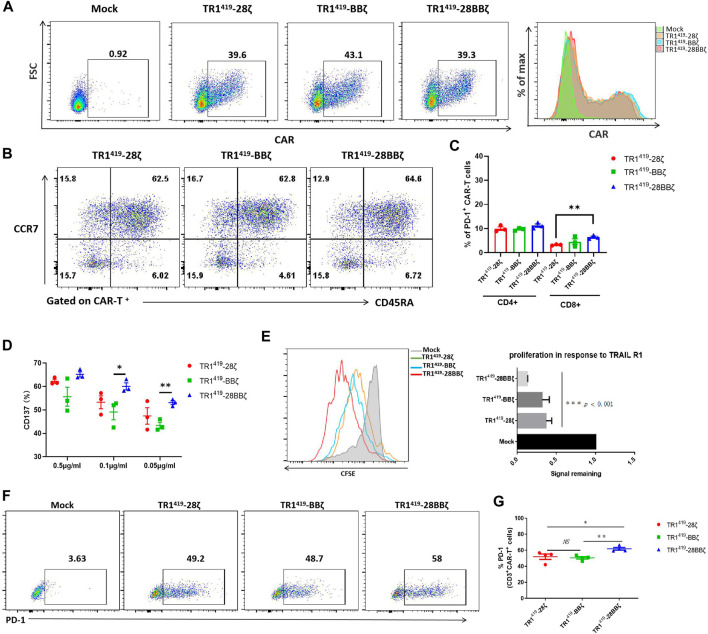
TR1^419^-28BBζ CAR-T cells exhibited functional advances upon antigen stimulation. **(A)** Untransduced Mock T cells or TRI^419^-28ζ TRI^419^-BBζ CAR-T cells were evaluated by flow cytometry for the TRI^419^ scFv expression. **(B)** Representative CAR-T cell phenotyping was plotted based on CD45RA and CCR7 expression. CD45RA^+^CCR7^+^: naive-like T cells; CD45RA^−^CCR7^+^: central memory T cells; CD45RA^−^CCR7^-^: effector memory T cells; and CD45RA^+^CCR7^-^: effector T cells. **(C)** Qualification of inhibitory molecule PD-1 expression on CD4^+^ CAR and CD8^+^ CAR-T cells on day 7 post lentiviral transfection. **(D)** Flow cytometric analysis of CD137 expression on TR1^419^ CAR-T cells cultured overnight with plate-bound recombinant human TRAIL-R1 at various concentrations. **(E)** TR1^419^ CAR-T-cell proliferation, indicated by division of CFSE, after cultured with SW480 for 7 days was assessed by flow cytometry. The signal remaining value corresponded to the mean fluorescence intensity of the CFSE of mock T cells. **(F–G)** The expression of PD-1 on TRI^419^ CAR-T cells was analyzed by FACS after 7 days of coculture with tumor cells. Data are given in the mean ± SD of three independent experiments, *p < 0.05, **p < 0.01.

In order to determine the functionally optimal CAR for the TR1^419^ scFv structural design, we generated three different TR1^419^ CARs with distinct intracellular compartment. Lentiviral vectors were constructed with the scFv region of TR1^419^, which were paired with the CD28, the 4-1BB, or the CD28-4-1BB co-stimulatory domain and combined with a CD3ζ activation domain (Supplementary Figure S1). Such vectors were transduced into the PBMCs derived from healthy donors to obtain the human TR1^419^-28ζ, TR1^419^-BBζ, and TR1^419^-28BBζ CAR-T cells, respectively. A flow cytometric analysis confirmed the similar surface expression levels of corresponding CARs on each type of T cells (Figure 1A). We checked for the phenotypes of these T cells and found that naive T cells constituted approximately 60% of each CAR-T cell populations (Figure 1B). There were no apparent variations in the different subtype proportions shown among the three types of CAR-T cells (Figure 1B). Moreover, we analyzed the exhaustion markers of these T cells, and no significant differences of PD-1, LAG-3 and TIM-3 expression were observed among the TR1^419^-28ζ, TR1^419^-BBζ, and TR1^419^-28BBζ CAR-T cells (Figure 1C, Supplementary Figure S2). These data demonstrated that there were no marked differences in the T cell phenotypic features between introducing the second or the third generation of TR1^419^-CARs.

Next, we examined the cellular activities of TR1^419^-28ζ, TR1^419^-BBζ, and TR1^419^-28BBζ CAR-T cells upon antigen stimulation. After 24 h of stimulation with different concentrations of plate-bound recombinant human TRAIL-R1 protein, the expression of CD137 was shown to be significantly higher in TR1^419^-28BBζ CAR-T cells at different antigen concentrations (Figure 1D). In addition, the T-cell proliferation was studied using CFSE staining, and the cell proliferation was analyzed by quantifying the fluorescent intensities of CFSE (cells with low CFSE peaks are proliferating cells) (Hadjadj et al., 2020). We found that the number of all three types of CAR-T cells was enlarged after cocultured with TRAIL-R1–positive SW480 cancer cells for 7 days (Figure 1E). Among them, TR1^419^-28BBζ CAR-T cells showed superior expansion capability compared with TR1^419^-28ζ and TR1^419^-BBζ CAR-T cells, and no significant difference was detected in cell proliferation between the 2 second-generation CAR-T cells (Figure 1E). A comparably higher level of PD-1 expression was found in TR1^419^28BBζ CAR-T cells than in TR1^419^-28ζ or TR1^419^-BBζ CAR-T cells after co-incubation with SW480, which was in line with the findings shown before that PD-1 was more frequently expressed by the TR1^419^-28BBζ CAR-transduced T cells (Figures 1F,G). Collectively, these results showed that the co-stimulatory domain of the TR1^419^-28BBζ CAR was associated with significantly higher levels of CAR-T cell activation and proliferation upon antigen stimulation.

### TR1^419^-28BBζ CAR-T Cells Exhibited Marked Cytolysis Efficacy Against TRAIL-R1–Expressing Tumor Cells

**FIGURE 2:**
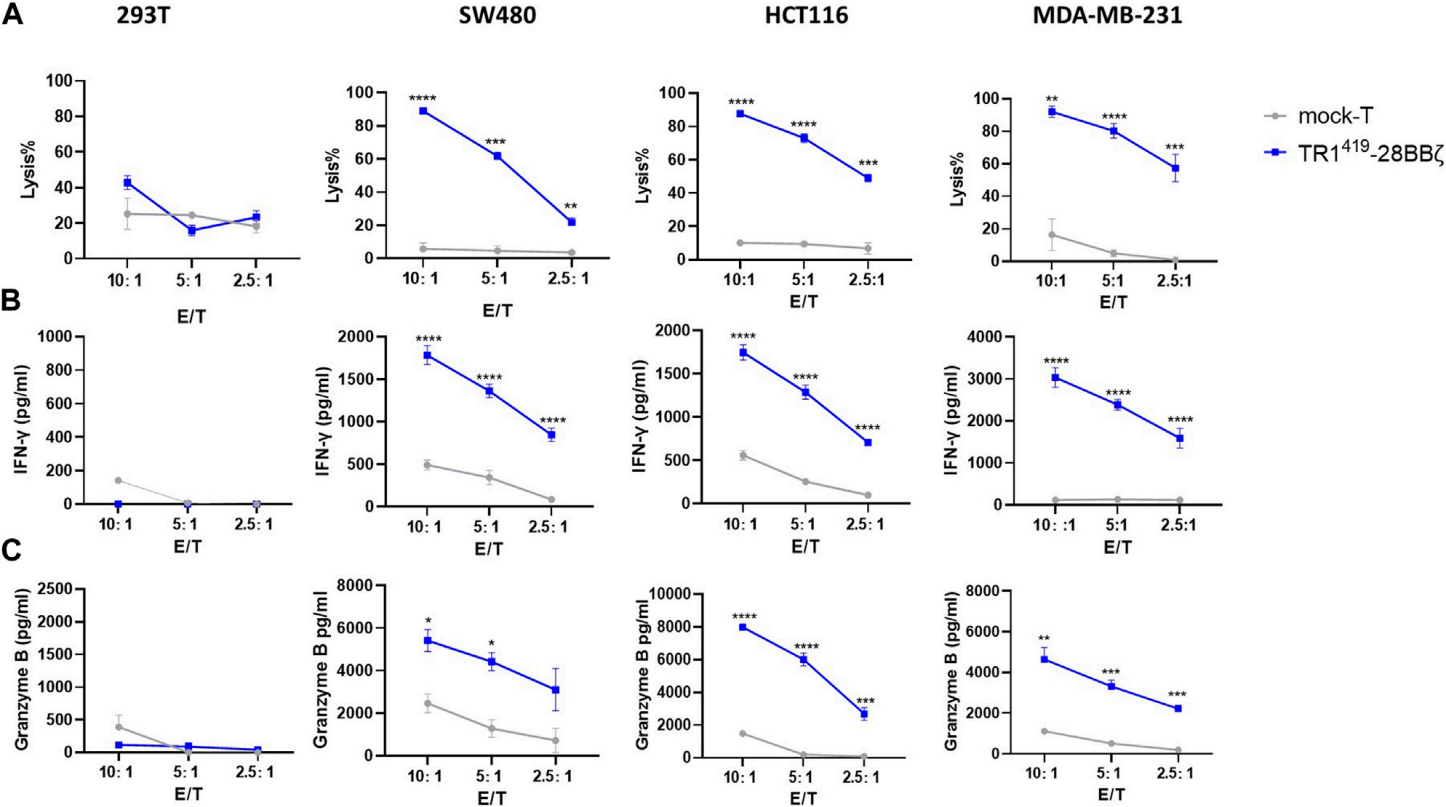
TR1^419^-28BBζ CAR-T cells induced significant cytolysis of TRAIL-R1–expressing tumor cells. **(A)** Cytotoxic activity of TR1^419^-28BBζ CAR–transduced T cells or mock T cells against TRAIL-R1–positive tumor cell lines was determined by calcein AM release–based assay. The effector cells were cocultured for 6 h with target cells at E:T ratio of 10:1, 5:1, and 2.5:1, respectively. IFN-γ **(B)** and granzyme B **(C)** production by TR1^419^-28BBζ CAR-T cells or mock T cells when cocultured with the indicated cells for 24 h was deducted by ELISA. Data are given in the mean ± SEM of three separate experiments. *p < 0.05, **p < 0.01, and ***p < 0.001.

In order to study the cytotoxic efficacy of TR1^419^-28BBζ CAR-T cells against target tumor cells *in vitro*, we screened for a panel of TRAIL-R1–positive cancer cell lines (Supplementary Figure S3). Results from the calcein AM release–based cytotoxic cell assay showed that TR1^419^-28BBζ CAR-T cells exhibited profound potency in killing TRAIL-R1–expressing SW480, HCT116, or MDA-MB-231 cells than the mock T cells at all E:T ratios (Figure 2A). Such cytolytic effects were accompanied by significantly increased IFN-γ and granzyme B secretions (Figures 2B,C). In comparison, the TRAIL-R1–negative 293T cells showed no susceptibility toward TR1^419^-28BBζ CAR-T cells in the coculture system, as no significant differences in cell lysis or effector cytokine secretions were observed comparing with the mock T group. Also, we found that TR1^419^-28ζ CAR-T cells could effectively kill SW480 and HCT116 tumor cells, with the efficiency similar as TR1^419^-28BBζ CAR-T cells, while the cytolytic efficacy of TR1^419^-BBζ was comparatively lower than that of other CAR-T cells against HCT116 cells (Supplementary Figure S4). These data demonstrated that the engineered TR1^419^-28BBζ CAR-T cells exhibited high functional efficacy against multiple tumor cells expressing the surface receptor TRAIL-R1.

### The ScFv Region From the TR1^419^ CAR on Jurkat Cells Could Induce Target Tumor Cell Apoptosis

**FIGURE 3:**
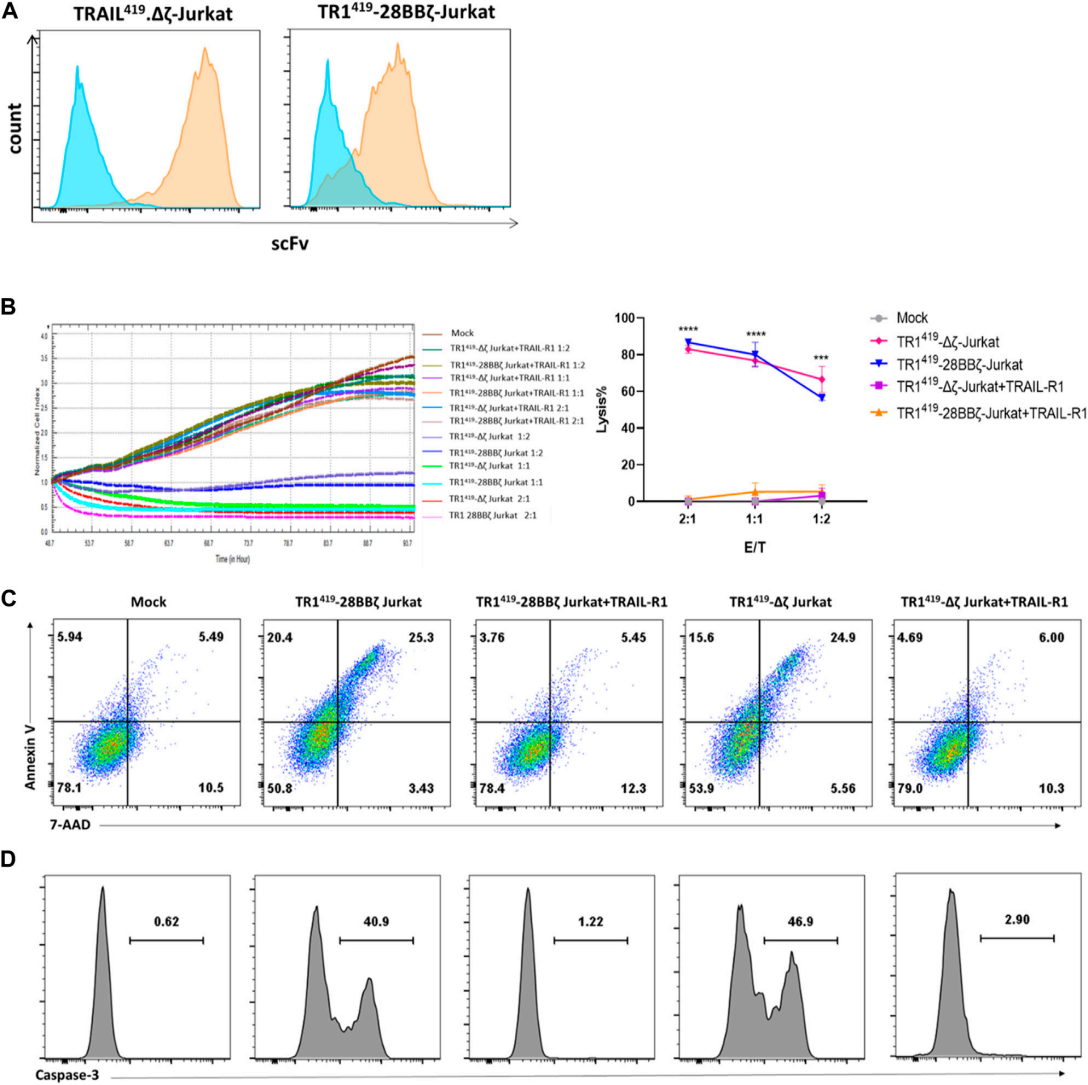
scFv from TR1^419^ CAR-mediated tumor cell apoptosis by binding with TRAIL-R1. **(A)** The expressions of TR1^419^-28BBζ in Jurkat cells were detected by flow cytometry. **(B)** Cytotoxicity of TR1^419^-28BBζ- and TR1^419^Δζ-Jurkat cells against SW480 in the absence or in the presence of soluble TRAIL-R1 protein was determined by RTCA. **(C)** SW480 cocultured with TR1^419^-28BBζ-and TR1^419^Δζ-Jurkat cells for 2 h before subjected for FACS to identify apoptotic cell populations (Annexin V+/7-AAD− and Annexin V+/7-AAD+). **(D)** The expression of caspase-3 in SW480 was analyzed by flow cytometry, after incubation with effector cells for 2 h. Data are represented in mean ± SEM of triplicate wells, ***p < 0.001.

To access the TRAIL-dependent cytotoxic effect with the TR1^419^-28BBζ CAR-T cells, we transduced the third-generation TR1^419^ 28BBζ CAR into Jurkat cells, which were unable to generate regular T cell cytolytic functions. In addition, we constructed a TR1^419^∆ζ lentiviral vector, carrying the truncated TR1^419^ CAR with no co-stimulatory domain or CD3ζ domain to completely blunt T-cell activation (Hayes et al., 2002; Yudushkin and Vale, 2010) (Supplementary Figure S1). The flow cytometry analysis confirmed that the expression of TR1^419^ scFv could be detected on the surface of both TR1^419^-28BBζ CAR- and TR1^419^∆ζ CAR–expressing Jurkat cells (Figure 3A). Target cell death was monitored using the RTCA based killing assay, and we found that TR1^419^-28BBζ and TR1^419^∆ζ-Jurkat cells could both efficiently induce SW480 tumor cell apoptosis in less than 24 h of coculturing. Furthermore, we utilized the soluble recombinant human TRAIL-R1-Fc proteins to block the scFv region expressed on both TR1^419^-28BBζ and TR1^419^∆ζ CAR-T cells, in order to confirm whether the extracellular domain of TR1^419^ CAR could induce tumor cell death. We found that co-treatment of such soluble TR1^419^ scFv blockers could completely abrogate the cytolytic effect of both types of CAR-Jurkat cells on the SW480 target cells (Figure 3B).

To determine the activation of the apoptosis pathway downstream of the target antigen, we performed the flow cytometric analysis for the expression of Annexin Ⅴ/7-AAD and caspase-3/cleaved caspase-3 in SW480 cells. It was shown that the percentages of apoptotic SW480 cells were similar as 45.7 and 40.5% for the TR1^419^-28BBζ-Jurkat cells and TR1^419^∆ζ-Jurkat cells cocultures, respectively (Figure 3C). These CAR-Jurkat cells induced levels of apoptosis were significantly higher than the control mock-Jurkat cells as well as the cocultures mixed with soluble target antigen recombinant proteins (Figure 3C). Similar results were found for Caspase-3 expression in SW480 cells (Figure 3D). Collectively, these results suggested that the scFv region from the TR1^419^ CAR played an independent role in the cytolytic effect of the CAT-T cells as it could mediate apoptosis in the TRAIL-R1–positive tumor cells.

### TR1^419^-28BBζ CAR-T Cells Exhibited Dual Antitumor Effects

**FIGURE 4:**
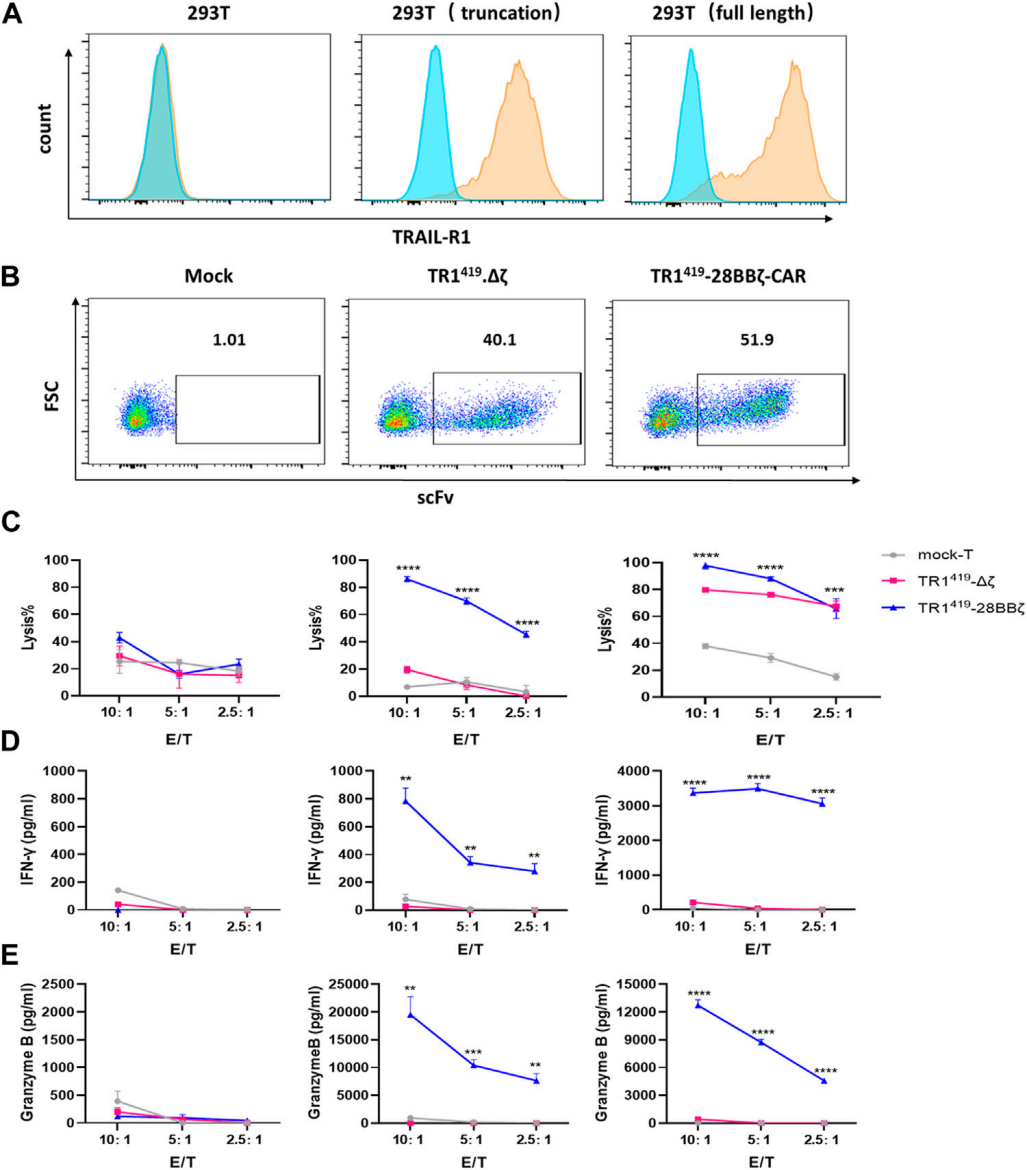
TR1^419^-28BBζ CAR-T cells mediated dual antitumor effects. **(A)** 293T cells were transfected with the lentiviral plasmids expressing the full length or truncated TRAIL-R1. Then the expression of both types of exogenous TRAIL-R1 in 293T cells was determined by flow cytometry. **(B)** Mock T, TR1^419^-28BBζ CAR-T cells, or TR1^419^Δζ-T cells were evaluated for the TR1^419^ scFc expression by flow cytometry. **(C)** Cytotoxic activity of TRI419-28BBζ CAR- and TR1^419^Δζ-T cells were determined against 293T; 293T expressing truncated TRAIL-R1 and 293T with the wild type TRAIL-R1, by RTCA assay. IFN-γ **(D)** and granzyme B **(E)** production by TR1^419^-28BBζ CAR-T cells or TR1^419^Δζ-T cells was detected by ELISA, after cocultured with the indicated target cells for 24 h. Data are represented in the mean ± SEM of three triplicate wells. *p < 0.05, **p < 0.01 and ***p < 0.001.

To further confirm TR1^419^-28BBζ CAR-T cells exhibited dual antitumor effects through death receptor TRAIL-R1–mediated apoptosis as well as T cell induced cytolysis, we established two target lines with 293T cells. 293T cells were exogenously introduced with the wild type receptor or a truncated TRAIL-R1 missing the death domain that was responsible for intracellular activation of the apoptosis pathway (Schneider et al., 1997) (Supplementary Figure S5). In parallel, we generated TR1^419^∆ζ-T cells by lentiviral transduction, to be used as a negative control for the TR1^419^-28BBζ CAR-T cells (Figures 4A,B). Then we performed cytotoxicity assays using TR1^419^-28BBζ CAR-T cells and TR1^419^∆ζ CAR-T cells as effector cells, cocultured with the 293T target system. For the 293T cells expressing the full-length TRAIL-R1, the most efficient level of cell killing was achieved with the TR1^419^-28BBζ CAR-T cells. Blocking the T-cell activation signal could reduce the overall level of cell lysis only at higher E:T ratios. In 293T cells with modulations on the integrity of the death receptor pathway, TR1^419^-28BBζ CAR-T cells could induce cell lysis in high efficiency, but such cytotoxic effect was completely abolished when T-cell activation was interrupted in the TR1^419^∆ζ CAR-T cells (Figure 4C). Because truncating the DD intracellular region of the antigen would not affect the CAR recognition, the production of IFN-γ and granzyme B were remained at a relatively high level in both 293T (full-length) and 293T (truncation) cells, whereas they were dropped to barely detectable levels when cocultured with TR1^419^∆ζ CAR-T cells (Figures 4D,E). These results suggested that TR1^419^ CAR-T cells exhibited cell killing efficacy not only by inducing T cell–mediated cytolysis of tumor cells but also by promoting tumor cell death through activation of the TRAIL-R1–dependent apoptotic pathway.

## Discussion

In the present work, we demonstrated that the third-generation TR1^419^-28BBζ CAR-T cells showed functional advances *in vitro*. They exhibited potent dual antitumor activities against various types of cancer cells by mediating target cell apoptosis through activation of the death receptor–dependent pathway as well as T cell–mediated cytolysis upon binding of the TRAIL-R1 antigen presented on the target cell surface. This study offers a strategic way for the design of CARs that may ultimately benefit the treatment of solid tumors.

Given the clinically presented difficulty for CAR-T cell therapy to reach desirable efficacy against solid tumors, combination therapy of CAR-T based ACT with other treatment regimens have been studied (Tong et al., 2020; Wang et al., 2020). The CAR-T cells shown in our study were designated to recognize-and-activate an independent mechanism to induce cellular apoptosis. Comparing to the other typical treatment methods used to augment CAR-T–based antitumor effect, such as chemotherapy and radiotherapy, this apoptotic way of target cell death was rather mild for inducing localized immune cell damage (Sims, 2012). Thus, the TR1^419^-28BBζ CAR, or similar designs for improving overall CAR-T cytolytic efficacy, might provide a potential suitable target to reduce the limitations associated with the clinical application of CAR-T cell therapy against TRAIL-R1–positive solid tumors.

As for the target antigen selection, TRAIL-R1 has been the focus of anticancer treatment during the last decade because of its preferentially increased expression in a large number of tumors (Satta et al., 2019; Zhang et al., 2019; Park et al., 2020). Although therapeutic approaches using the natural ligand TRAIL have been demonstrated with little antitumor efficacy, recent studies evidenced that effective TRAIL-based therapies could be achieved by ligand modifications, that is, additional crosslinking to TRAIL-R1 antibodies (Hao et al., 2016). Our data confirmed that the TR1^419^-28BBζ CAR constructed with the scFv from the TR1^419^ antibody could directly mediate tumor cell apoptosis by binding with its antigen without the need of additional crosslinking structures. This suggests the possibility that the collected structure of TR1^419^-28BBζ CAR may be as functionally sufficient as a “modified ligand” to induce apoptosis in the target cells through effective aggregation of the cognate death receptor, which has been shown to be the key of sufficient apoptotic pathway activation and consequential cell death previously reported (Voigt et al., 2014; Dufour et al., 2017). Also, we have been proven that CAR-Jurkat cells have no ability to mediate antigen-positive target cytolysis (Supplementary Figure S6). The fact that the TR1^419^-28BBζ CAR could equip Jurkat cells as well as PBMCs to promote effective tumor cell apoptosis highlighted the functional significance of this structural design in TRAIL-based CAR-T cell therapies.

Another functional determinant for the optimal structure of a CAR is the co-stimulation domain. It has been reported that the second- and third-generation CAR-T cells may exhibit phenotypic and functional differences, corresponding to the selection of target antigen and the design of scFv structure (Boroughs et al., 2019). In the process of determining the appropriate CAR for our own TR1^419^ scFv structure, we found that the co-stimulatory domain in the 28BBζ CAR conferred improved selectivity for lower tumor antigen density and persistent proliferative potential upon antigen stimulation, comparing to the 28ζ or the 4-1BBζ CARs. Certainly, further experiments are needed to test these features. These features were accompanied with only slightly enhanced target cell killing for TR1^419^-28BBζ CAR-T cells within a relatively short time window, suggesting that these three CARs might be interchangeable in the fine-tuning of mechanism studies. However, PD-1 expression was found at comparatively higher levels in TR1^419^-28BBζ CAR-T cells than the other 2 second-generation CAR-T cells, both before and after antigen encounter, which might possibly lead to increased exhaustion (Rafiq et al., 2018). This raised the concern for the potency and persistence of TR1^419^-28BBζ CAR-T cells upon repeated TRAIL-R1 stimulation and/or for long time antigenic exposure (Saleh et al., 2020). In addition, future *in vivo* study may provide important information regarding the possibility of learning about some side-effects in a living organism, turning their results more representative to the clinical situation than those obtained *in vitro*, which may better help us in fine-tuning such CAR-T cell–based immunotherapeutic approaches.

Another essential aspect to be addressed with the adoptive transfer of CAR-T cells is the consequence of potential target recognition outside the context of cancer. Particularly for our TR1^419^ CAR-T cells, the expression level of the antigenic receptor protein on the target cell surface might greatly impact the potential off-target effects. In cells exhibiting no TRAIL-R1 expression, which was represented by HEK-293T cells in the current study, treatment using TR1^419^-28BBζ CAR-T cells were associated with bare minimum levels of cytotoxicity or secretion of effector cytokines, thus highlighting the target specificity of such effector cells (Voigt et al., 2014). It has been reported that the liver actually exhibited high tolerance to the TRAIL-R1 antibody–mediated killing, though the specific mechanisms remain unknown (Ma et al., 2017). One possibility might be that the comparatively low levels of TRAIL-R1 expression on normal tissues might limit the receptor aggregation upon the binding of ligand mimics, thus restricting the activation of the downstream apoptotic pathway. Comprehensive studies are definitely needed to determine the tumor specificity and/or potential off-target effects of TR1^419^28BBζ CAR-T cells. Nevertheless, together with new emerging technologies, for instance, by adjusting the CAR affinity, the drawback of non-target cytotoxicity associated with CAR-T therapies might be further circumvented and optimized.

In conclusion, we presented the third-generation TR1^419^28BBζ CAR-T cells with dual cytotoxic efficacy against various types of tumor cells, with high sensitivity and persistent proliferative capability upon antigen stimulation. This study provides a novel strategy for the design of CARs and an effective candidate for the adoptive CAR-T cell therapy targeting the TRAIL-R1–positive tumors.

## Data Availability

The original contributions presented in the study are included in the article/Supplementary Files; further inquiries can be directed to the corresponding author.
