# Supplementary figures and images for: TRAIL-R1-Targeted CAR-T Cells Exhibit Dual Antitumor Efficacy
Source: Front Mol Biosci. 2021 Dec 20;8:756599. doi: 10.3389/fmolb.2021.756599 (PMC8721281; doi:10.3389/fmolb.2021.756599)

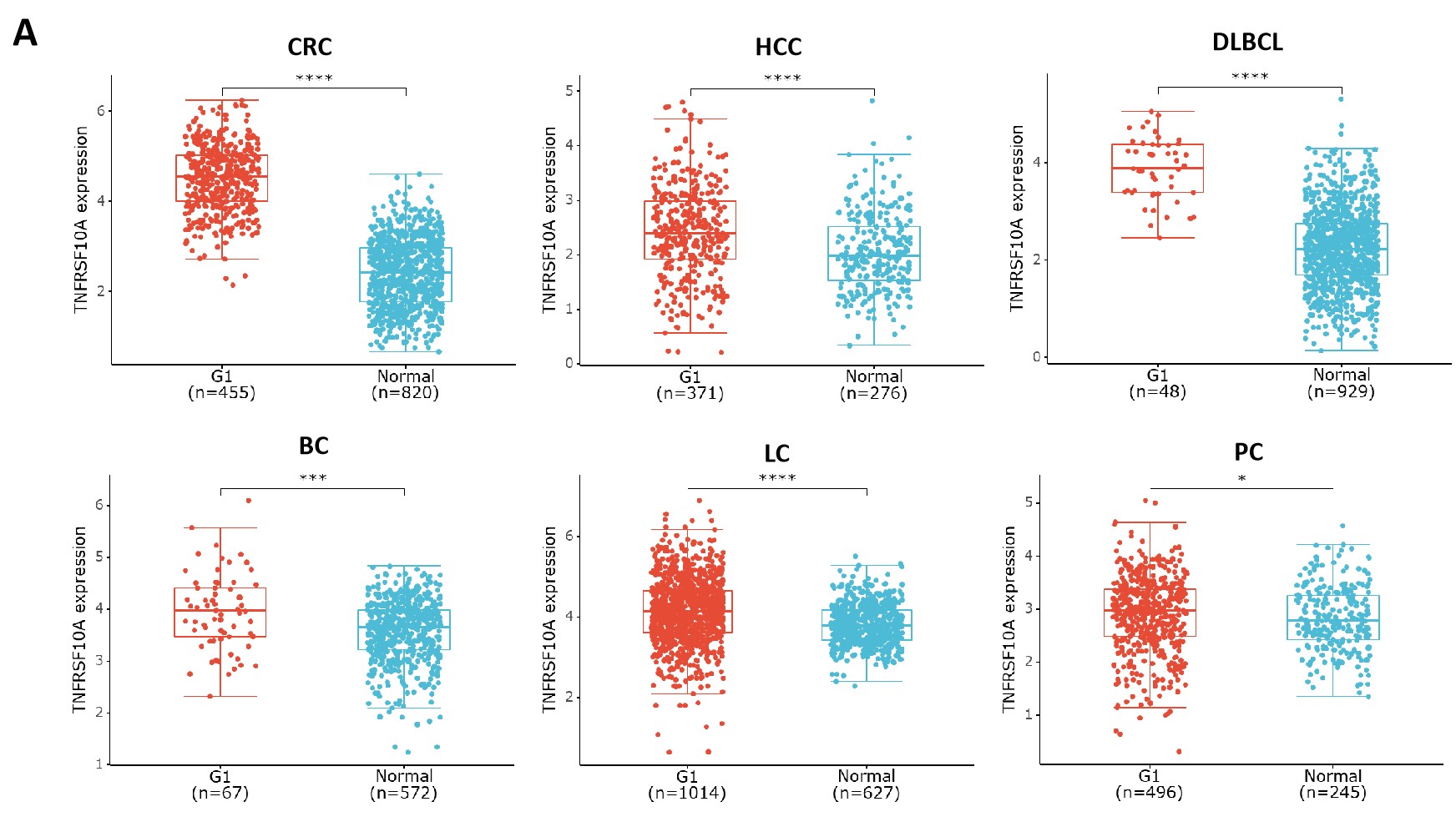

Supplement: Supplementary file 1 [file Image3.jpg]

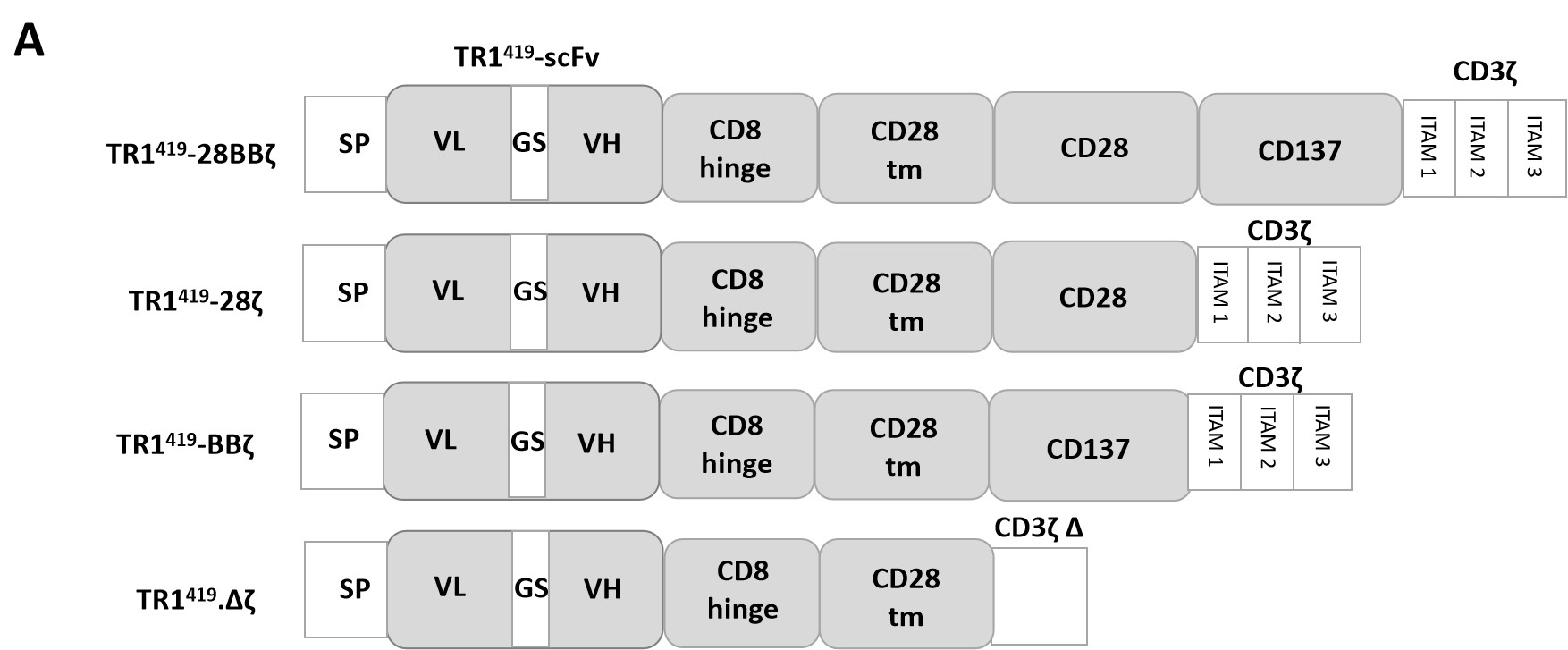

Supplement: Supplementary file 2 [file Image1.jpg]

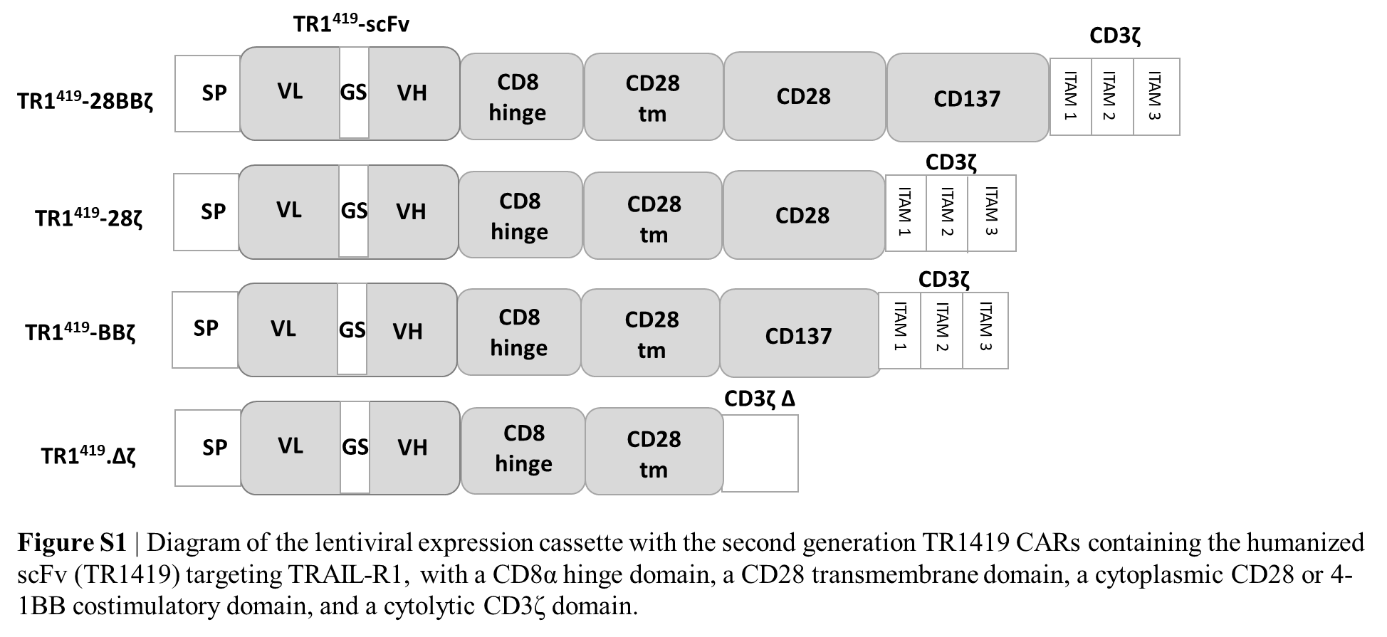


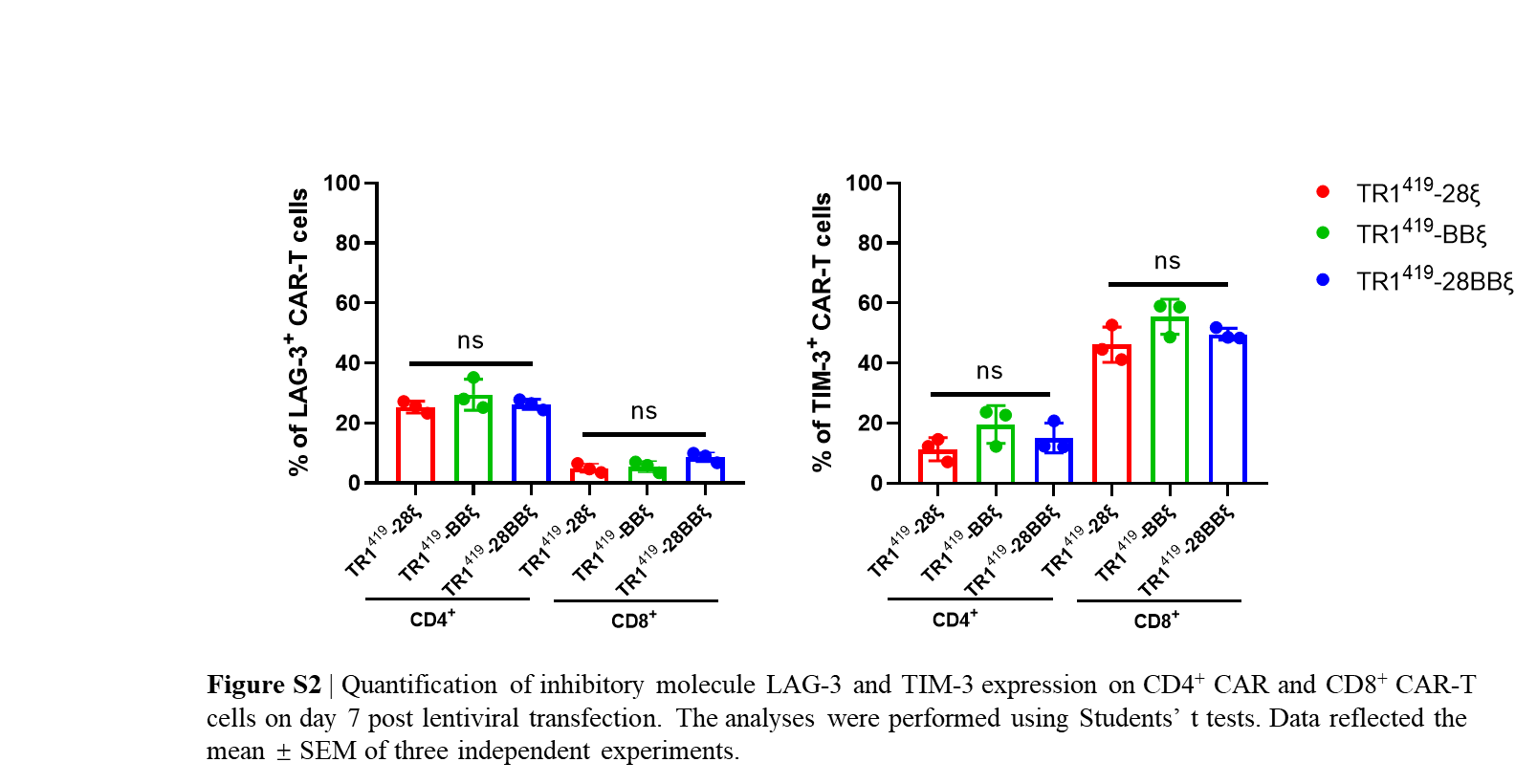


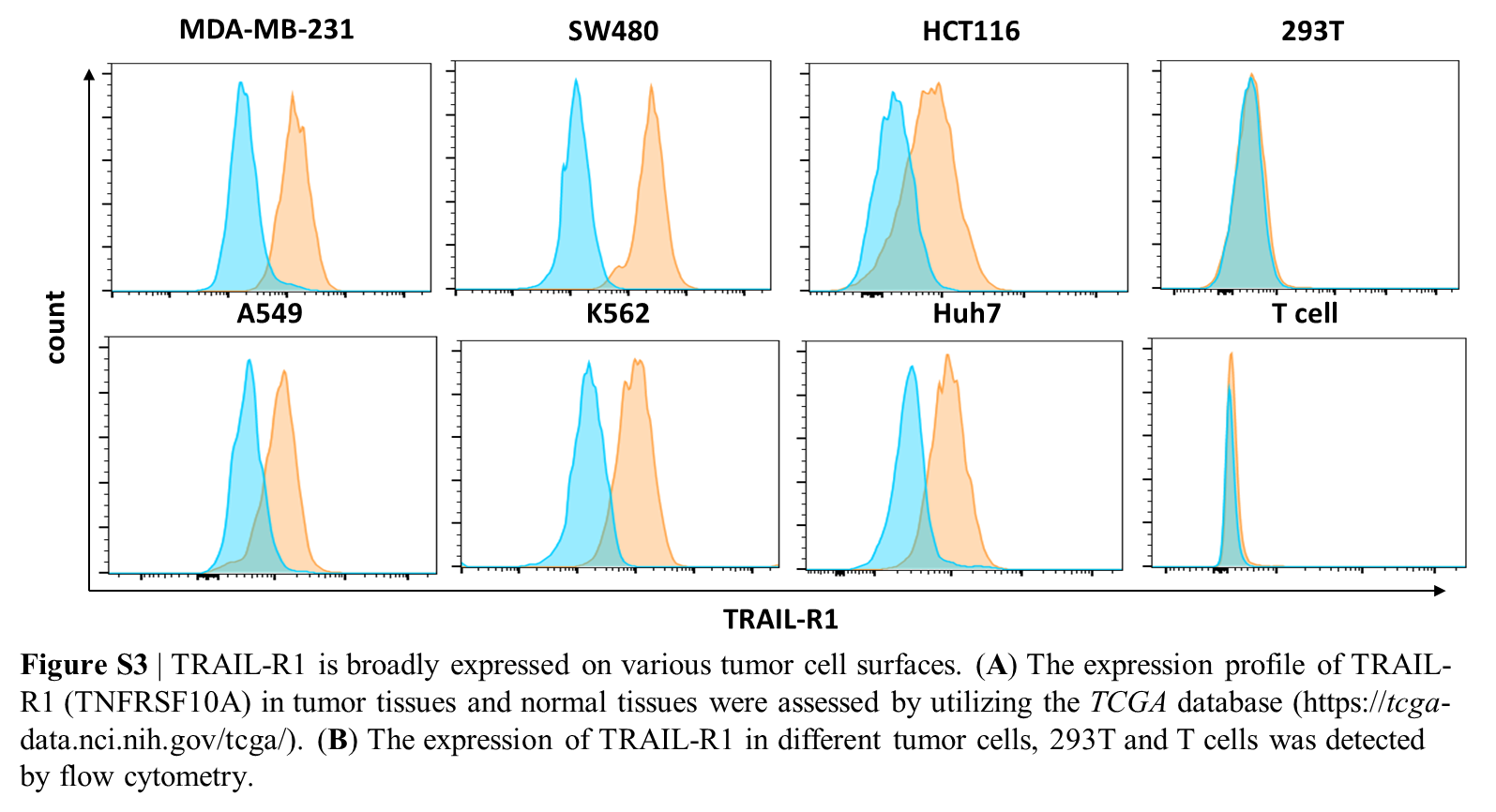


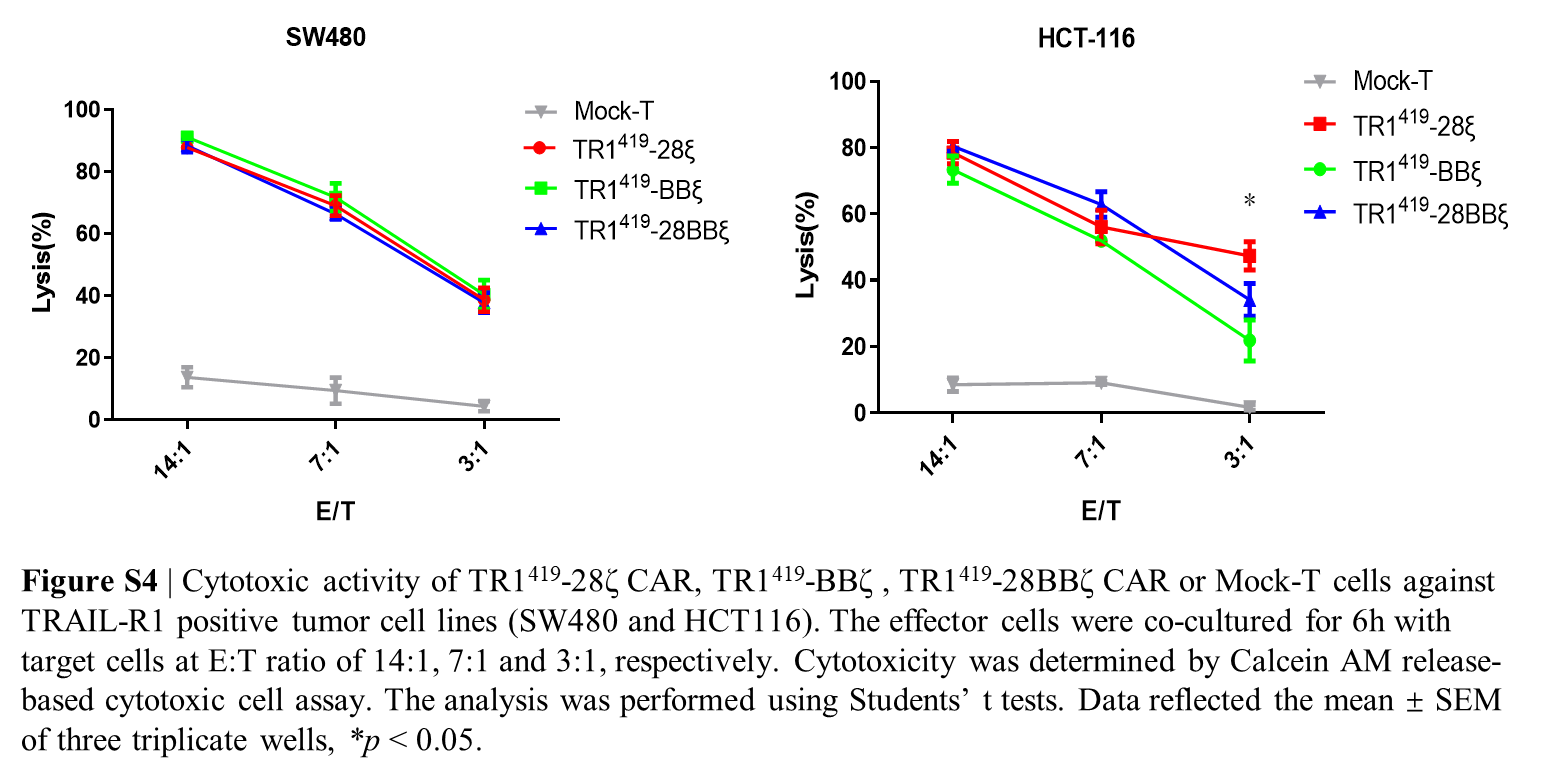


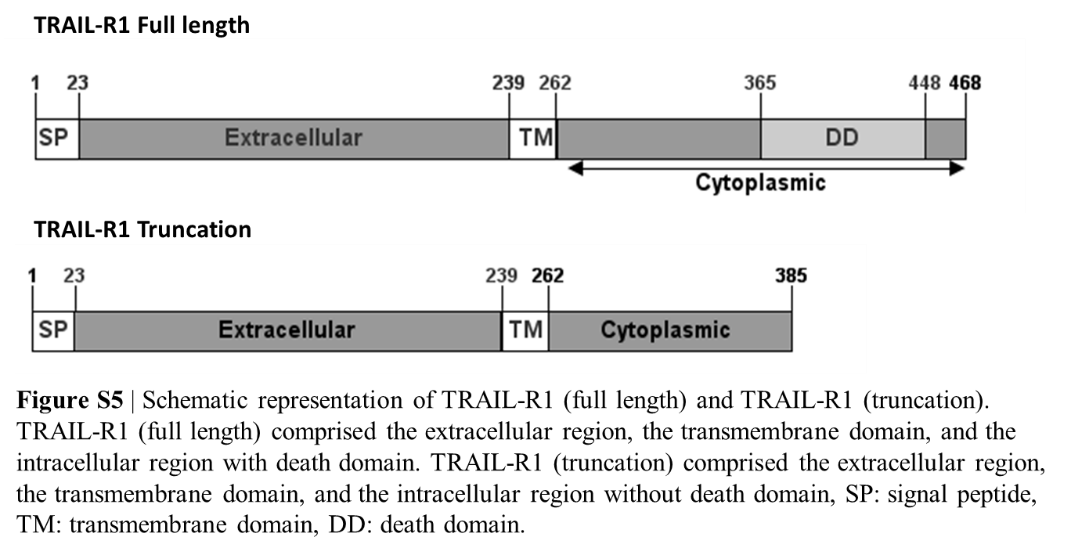


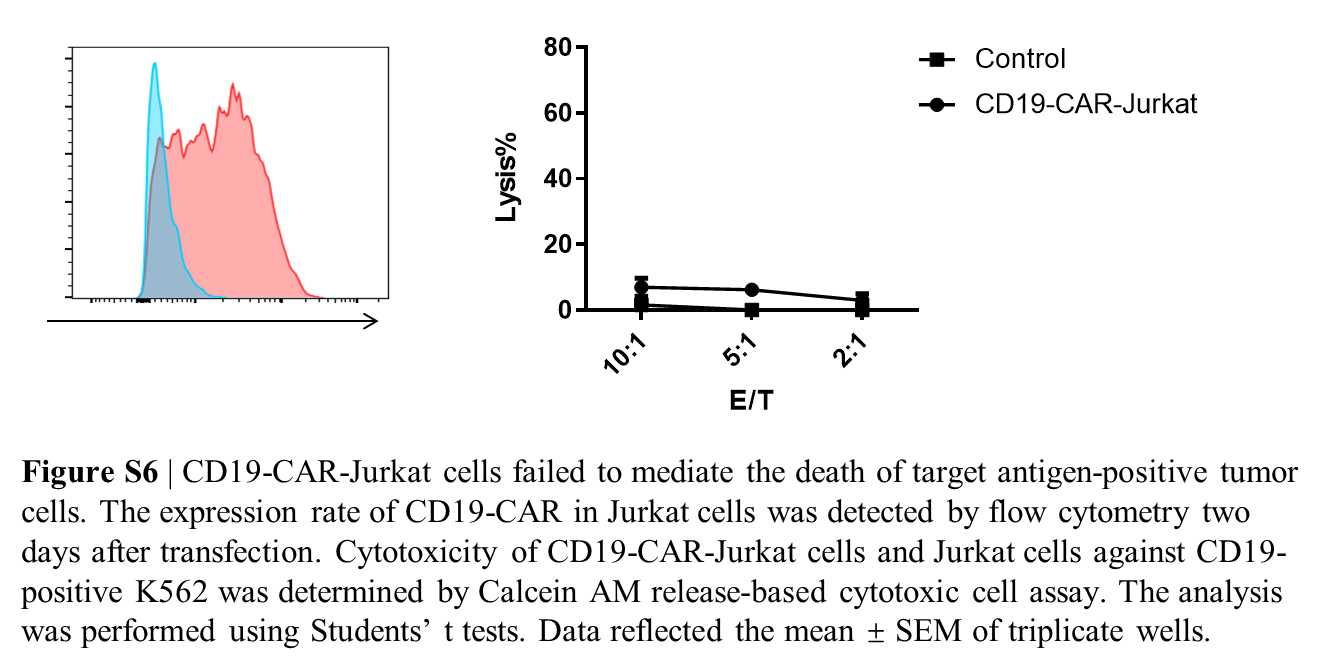

Supplement: Supplementary file 3 [file DataSheet1.docx]
